# Acute Zonal Occult Outer Retinopathy: Vision Loss in an Active Duty Soldier

**DOI:** 10.1155/2013/240607

**Published:** 2013-03-30

**Authors:** Courtney M. Crawford, Bruce A. Rivers, Mark Nelson

**Affiliations:** ^1^Blanchfield Army Community Hospital, 650 Joel Drive, Fort Campbell, KY 42223, USA; ^2^Winn Army Community Hospital, 1061 Harmon Avenue, Fort Stewart, GA 31315, USA; ^3^Madigan Army Medical Center, 9040 Fitzsimmons Dr., Fort Lewis, WA 98433, USA

## Abstract

*Objective*. To describe a case of acute zonal occult outer retinopathy (AZOOR) in an active duty patient. *Methods*. In this paper we studied fundus photographs, optical coherence tomograph, Humphrey visual field 30-2, fundus autofluorescence images, fluorescein angiograms, and electroretinography. *Results*. Exam findings on presentation: a 34-year-old American Indian female presented with bilateral photopsias, early RPE irregularity, and an early temporal visual field defect. Progression RPE damage and visual field defect along with ERG findings support final diagnosis of AZOOR. *Conclusion*. AZOOR may initially be identified as a broader category of disease called the “AZOOR complex of disorders”. Specific visual field defects, ERG results, and clinical exam findings will help distinguish AZOOR from other similar disorders.

## 1. Introduction

Acute zonal occult outer retinopathy (AZOOR) was first described by Gass who noted in these patients a loss of outer retinal function, minimal fundus changes, irreversible ERG abnormalities, and permanent visual field loss [1]. Other conditions associated with AZOOR that Gass categorized as the “AZOOR complex of disorders” include idiopathic blind-spot enlargement syndrome, MEWDS, PIC, acute macular neuroretinitis, and multifocal choroiditis [1–3].This condition has not been reported in an active duty military or American Indian population.

## 2. Case Report

**Figure 1:**
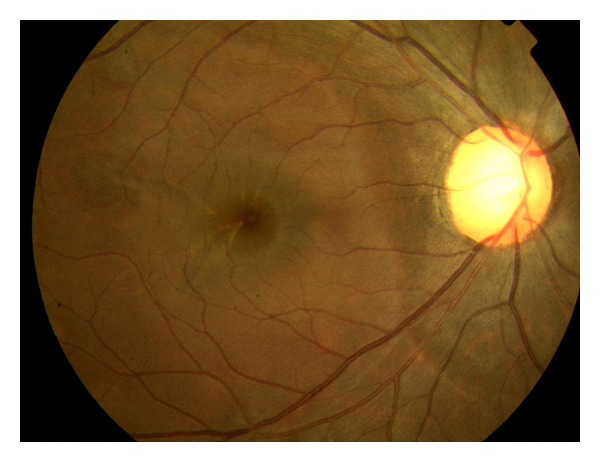
Color fundus photo (right eye).

**Figure 2:**
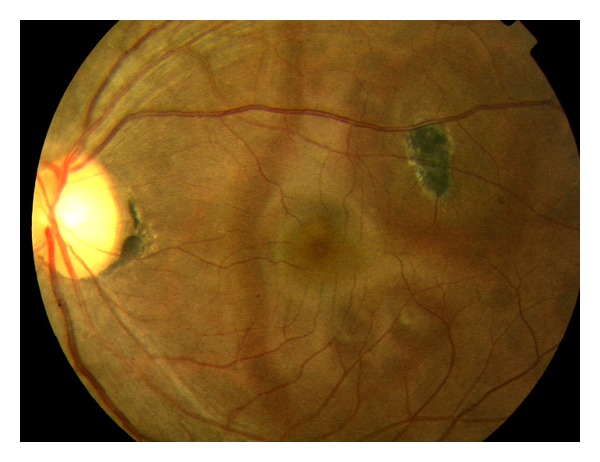
Color fundus photo (left eye).

A 33-year-old active duty American Indian female initially presented with bilateral photopsias and temporal visual field defects. She was referred from a community ophthalmologist with the diagnosis of a “possible white dot syndrome”. She has a history of high myopia (−6.75D OD/−7.00D OS).Her BCVA was 20/20 OD and 20/30 OS. The anterior segment ophthalmoscopic findings were normal bilaterally. Posterior segment evaluation showed that no vitritis, scalloped and circular RPE defects OU, and a known previous chorioretinal scar OS (Figures 1 and 2). 

**Figure 3:**
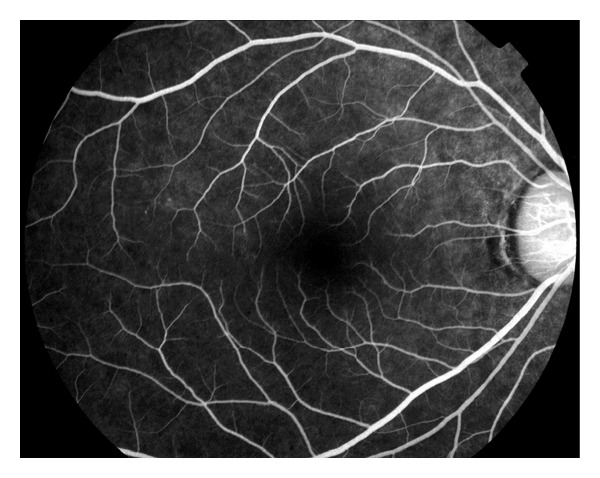
Fluorescein angiography (right eye).

**Figure 4:**
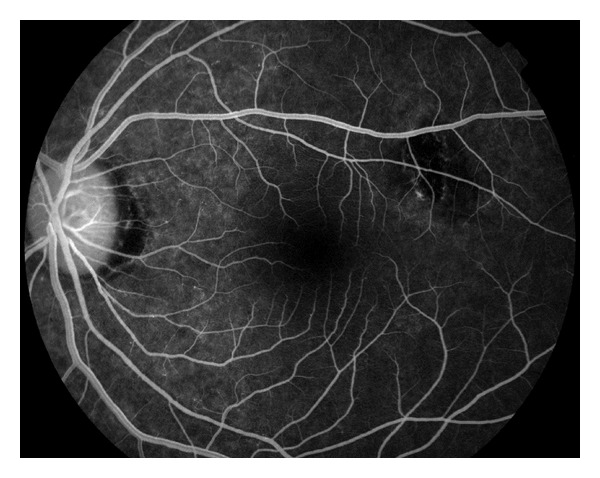
Fluorescein angiography (left eye).

Humphrey visual field 30-2 revealed enlarged blind spots and temporal scotomas of both eyes (Figures 3 and 4). The temporal scotomas correlated with the area of RPE atrophy and the patients stated location of photopsias. The electroretinography (ERG) showed the cone and flicker response was reduced in both eyes. Optical coherence tomography showed a normal IS-OS line in the macula but loss of the IS-OS line in the paramacular areas that corresponded to the area of defective visual fields. The retinal thickness was normal despite the abnormal IS-OS junction. Laboratory studies to rule out syphilis, cytomegalovirus, herpes zoster, and simplex were negative. Antinuclear antibody, rheumatoid factor, and HIV were negative. Imaging studies to include chest X-ray and magnetic resonance imaging of the brain were normal. Over the course of 12 months the unchanged BCVA, visual field, OCT, and RPE defects pointed toward a diagnosis of AZOOR. Following the results of the ERG (showing global defective photoreceptors) the diagnosis of AZOOR was more definitive. 

## 3. Discussion

**Figure 5:**
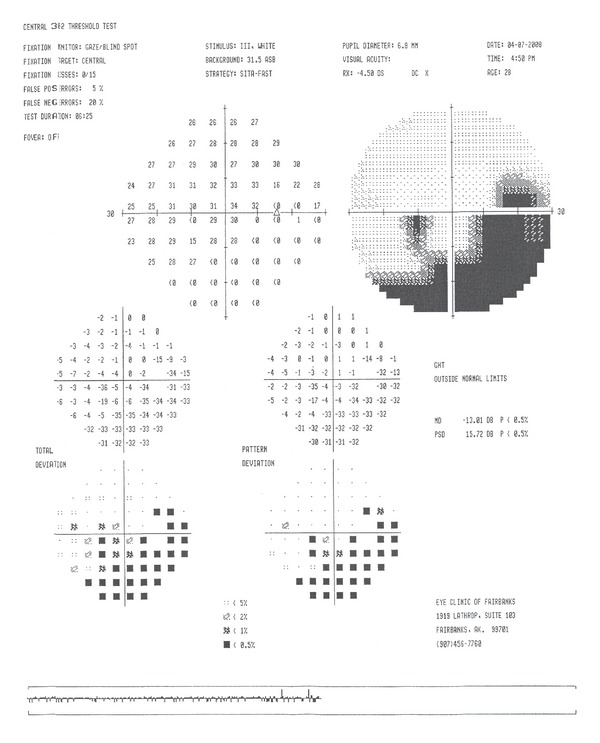
Visual field 30-2 (right eye).

**Figure 6:**
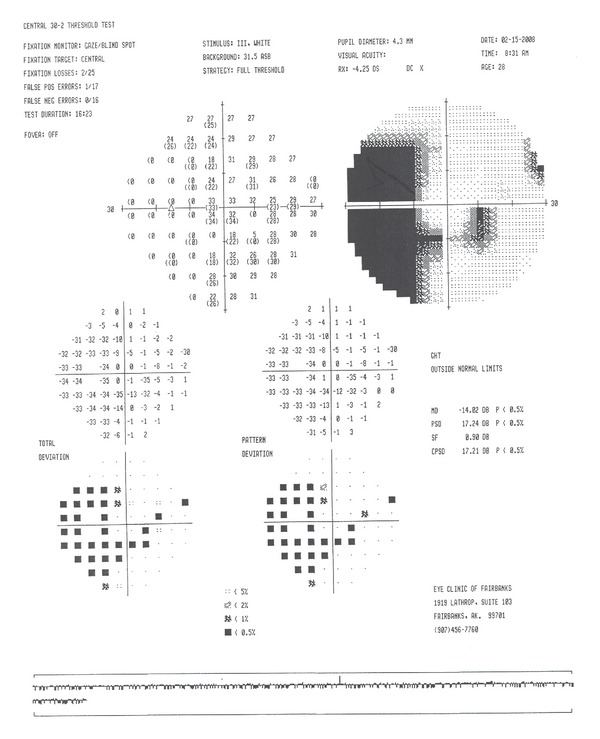
Visual field 30-2 (left eye).

AZOOR is a rare eye disease, marked by typical characteristics of photopsias, visual field defects, abnormal ERG, and subtle changes to the fundus. It is often found in young myopic females, similar to other white dot syndromes of MEWDS and PIC [4, 5]. It was first described by Gass, who described AZOOR as minimal fundus or fluorescein angiographic changes, loss of zones of outer retinal function, irreversible ERG abnormalities, and permanent visual field loss [1]. Late disease changes include atrophy of the photoreceptors, narrowing of arterioles, and pigment migration in a bone spicule pattern [3].Pathologically, the primary lesion in AZOOR is a photoreceptor outer segment degeneration.Often the pathology and decreased vision are present in a unilateral presentation; however concomitant involvement of the fellow eye is observed in some individuals [5] (Figures 5 and 6).

Despite the focal nature of the AZOOR clinically, there is electrophysiological evidence of global and generalized dysfunction affecting both the cone system and the retinal pigment epithelium [6]. Such a pattern is not observed in other white dot syndromes, which may serve to distinguish AZOOR from others [7]. Consistent with photoreceptor dysfunction, our patient displayed reduced a-wave amplitudes on ERG. Typical of AZOOR, the 30 Hz flicker response was reduced. An electrooculogram was not performed; however most reports indicated a reduction of the light rise of the EOG to be consistent with AZOOR [5, 8].

The visual field pattern loss in AZOOR is most frequently temporal and complete. Initially, the visual field loss is progressive and then it becomes persistent [4]. Occasional visual field loss includes enlargement of the blind spot, similar to other white dot syndromes (i.e.: MEWDS). Patients will frequently correlate the location of the photopsias with the major zone of field loss. Not only did our patient's visual field worsen then remain constant, she also consistently reported the photopsias to correlate with the area of her visual field defect.

The etiology of AZOOR remains unknown but a viral versus an inflammatory cause is suspected. The global electrophysiological abnormality may favor an inflammatory etiology over a viral cause [6]. Viral-mediated disorders such as MEWDS, PIC, and macular neuroretinitis accompany focal retinal dysfunction and are not associated with an abnormal full field ERG. Francis et al. proposed that because half of the patients in their study had suffered a preceding episode of MEWDS or PIC, it is possible the AZOOR is an autoimmune process triggered by a previous inflammatory event [6].

No treatment has been shown to effectively treat AZOOR. While one-third of patients may develop recurrence and portend a poorer visual outcome, the majority of patients have one episode with good visual recovery. According to Gass' series of patients 88% retain vision of 20/40 or better once the disease has subsided. In Gass' same patient population, improvement of the patient's vision occurred 6 months from initial presentation [1, 9].
